# Sustainable Employability of Emergency Nurses: The Effects of Precarious Work and Mental Toughness on Capabilities and Mental Health

**DOI:** 10.1155/2023/8840756

**Published:** 2023-05-26

**Authors:** Neil B. Barnard, Sebastiaan Rothmann, Leon T. de Beer, Welma Lubbe

**Affiliations:** ^1^Optentia Research Unit, North-West University, Vanderbijlpark Campus, Vanderbijlpark, South Africa; ^2^WorkWell Research Unit, North-West University, Potchefstroom Campus, Potchefstroom, South Africa; ^3^Department of Psychology, Norwegian University of Science and Technology, Trondheim, Norway; ^4^NuMIQ Research Focus Area, North-West University, Potchefstroom Campus, Potchefstroom, South Africa

## Abstract

Studying the sustainable employability of emergency nurses is important, given the precarious environment in which they work. This study used a cross-sectional survey of 204 emergency nursing professionals to investigate their sustainable employability in a South African context from the perspectives of precarious work, mental toughness, capabilities, and mental health. The Precarity Position Profile, Mental Toughness Questionnaire-Short Form, Capability Set for Work Questionnaire, and Flourishing-at-Work Scale-Short Form were administered. Three precarious work dimensions negatively predicted emergency nurses' capabilities. Significantly, precarious work conditions and professional development were associated with most work capabilities. Emergency nurses' capability set positively affected their mental health, with mental toughness moderating the effect of poor salary (a component of precarious work) on capabilities. Precariousness regarding salary, work conditions, and professional development affected emergency nurses' mental health indirectly and negatively through a poor capability set, while mental toughness indirectly and positively affected their mental health through a strong capability set.

## 1. Introduction

Precarious work is a global concern [1]. It is rising, resulting in adverse effects for the individual, workplace, and community [2, 3]. Pervasive uncertainty, characterised by dependence on others and vulnerability to others, is the core of precarity [4]. Therefore, work that exposes individuals to social and economic risk due to poor remuneration, job insecurity, and a lack of workplace protection can be considered precarious [5]. The work of emergency nurses (“emergency nurse” refers to any nurse (professional or lower categories) working in the emergency department of a hospital providing Level 1 or Level 2 trauma care) is precarious, with potential adverse mental health outcomes [6, 7]. Significantly, precariousness extends beyond poverty, unemployment, and poor employment conditions. Some of the demands contributing to the precariousness of emergency nurses include violent and abusive patients and families, treatment uncertainty [8], unfavourable management styles, a lack of appropriate support systems [9], and changes in the work environment, such as the implementation of complex information technology systems [10]. In addition, the SARS-CoV-2 outbreak, resulting in a global pandemic (COVID-19), placed a burden on emergency nurses due to the influx of patients and the rapid and continual changes in processes, reduced contact with patients, families, and colleagues, visitor and caretaker restrictions, lockdowns, and distancing requirements [11]. Furthermore, poor salaries and working conditions and increased job insecurity result in a precarious workplace that puts the sustainable employability of emergency nurses at risk [9, 11].

The quality of emergency nurses' working life is vital for sustainable development. The United Nations (UN) and International Labour Organisation [12] have argued its importance in the UN's Sustainable Development Goal 8 (SDG 8: Promote sustained, inclusive, and sustainable economic growth, full and productive employment, and decent work for all). Present-day workers regard the value of work as a significant aspect of the quality of their working life and sustainable employability [13, 14]. A key aspect of sustainable employability (SE) is that emergency nurses can achieve tangible opportunities through their capabilities during their working lives [13]. Consequently, their health and well-being are safeguarded, and they can contribute to society now and in the future. To exploit these opportunities, emergency nurses must have an enabling work environment and the attitude and motivation to take advantage of opportunities.

The quality of work continues to be transformed by forces such as globalisation, income inequality, organisational restructuring, eroding social safety nets, and digitalisation [1, 15]. Pfeffer [16] suggests that work environments adversely affect individual well-being and organisational performance, which may worsen because work is becoming increasingly precarious. Precarious work involves uncertainty regarding the work itself and how to cope with unexpected consequences due to a lack of social power and resources. Specifically, precarious work puts individuals' SE at risk because it affects their security [17], psychological safety [18], and mattering [19], which results in poor mental health outcomes. Therefore, it is critical to study how the quality of individuals' work relates to their mental health [6].

Emergency nurses' sustainable employability holds implications for them personally, the hospital or institution where they are employed, and eventually society at large [15]. Precariousness among emergency nurses can result in poor mental health, which, in turn, can lead to them resorting to unfavourable behaviours (such as disengagement or withdrawal) to protect themselves [15]. Significantly, precarious work can have an impact on emergency nurses' work capabilities [20], affecting their mental health. For institutions to provide proper emergency care, they need capable nurses to uphold quality patient care while ensuring sustainable employability.

From a social justice perspective, emergency nurses need the freedom to choose from many opportunities to engage in work activities they consider meaningful. This perspective implies that well-being is unattainable without fairness [19]. The capability approach (CA) uses social justice as a framework for studying employees' well-being, suggesting that people need to have the freedom to choose a life they have reason to value [21, 22]. Although the effects of inner resources and external barriers (and their interaction) on individual behaviours are continuously debated, Sen [23] maintains that people need to feel secure and free to develop their capacities and take effective action [24]. The core elements of the CA [23, 25] are capabilities, which refer to what people can do and be, and functionings, which are the corresponding accomplishments.

Within the SE framework, personal resources such as mental toughness influence emergency nurses' ability to convert available resources into tangible opportunities. Emergency nurses' level of mental toughness can mitigate the effects of precarious work on their capability set and their ability to realise valued work outcomes [13]. Therefore, mental toughness is a psychological resource that emergency nurses can utilise to pursue valued goals when navigating challenges and stressful situations [26].

Precarious work (as an external constraint) and mental toughness (as a personal resource) can affect emergency nurses' work capabilities and mental health (as a functioning) [13]. Emergency nurses with precarious jobs and low mental toughness, who lack work capabilities, are expected to have suboptimal mental health. Furthermore, the effects of precarious work and mental toughness on emergency nurses are largely unexplored in the South African context. Thus, the present study aimed to investigate the sustainable employability of emergency nurses from the perspectives of precarious work, mental toughness, capabilities, and mental health at work.

## 2. Sustainable Employability

Emergency nurses require relevant work capabilities to function well in their unique work environment. In this regard, Van der Klink [27] points out that the conceptualisation of work, health, and employability lacks a focus on work values. The sustainable employability model [13] aims to address this shortcoming by building on the CA [21] and, subsequently, emphasising work values. Therefore, the sustainable employability model offers a framework for investigating emergency nurses' capabilities and functionings. Within this framework, it is essential to investigate what work outcomes emergency nurses consider necessary, whether the work context enables them to achieve these outcomes, and whether they are achieving them. Accordingly, the CA focuses on people's capabilities, that is, what they can ultimately do (activities) and be (states), and whether they present with the capabilities to engage in valued functionings [28].

### 2.1. Capabilities

The capability approach (CA; [21]) conceptualises well-being as an ability to achieve valuable states (through capabilities) rather than economic utilities. In this sense, for people to have a flourishing life, they require resources and freedoms to create equal opportunities [29]. The CA acknowledges and is sensitive to human diversity and socio-cultural contexts [28], emphasising capabilities and not merely resources and functionings [21, 23, 25]. Capabilities are the true freedoms people have in being who they want to be (beings or states) and doing what they consider valuable (doings or activities) [30]. A person's capabilities originate from the freedoms produced by institutions, companies, and social relations in converting available resources into tangible opportunities (known as a capability set) that can be used to realise valued achievements (i.e., functionings).

Conversion factors are critical elements within the CA framework, as people potentially differ in their ability to convert available resources into capabilities. Consequently, conversion factors have an impact on whether individuals can convert resources at their disposal into realised functionings. Conversion factors are classified into three categories: (a) personal conversion factors that are internal to the person (such as age, gender, and education); (b) social conversion factors that are social norms and cultures, ethnic profiles, gender roles, and power relations; and (c) environmental conversation factors that include climate and transportation [28].

Van der Klink et al. [13] argue that increasing workers' capabilities (i.e., their freedom to achieve work values) will improve their sustainable employability, resulting in increased resilience and well-being. Abma et al. [31] identify seven work capabilities: (a) using knowledge and skills; (b) developing knowledge and skills; (c) involvement in important decisions; (d) building and maintaining meaningful contacts at work; (e) setting own goals; (f) earning a good income; and (g) contributing to something valuable. The sustainable employability framework argues that a capability set includes individuals' abilities and opportunities to achieve work goals they have reason to value. Capabilities form part of emergency nurses' capability sets, depending on whether individuals perceive them as important, are enabled to use them, and achieve them. Conversion factors important among workers include: (a) organisational conversion factors, i.e., cultural aspects, power relations, shortage of personnel, and policies for self-management; (b) work conversion factors, i.e., social contacts, communication, workload, tasks, and schedules; and (c) personal conversion factors, i.e., experienced work stress, motivation, and the ability to achieve values informally within the company [32].

### 2.2. Precarious Work

Precarious work, low earnings, and insufficient employee representation are rising [3], which has an impact on employees' physical and mental health [33–35]. Precarious work shares some of its characteristics with other models of poor work such as underemployment [36] and the inverse of decent work [37]. However, such work differs from these models because of the focus on uncertainty, insecurity, and instability [6]. The ILO's decent work agenda emphasises the importance of productive and sustainable work, enhancing social protections for workers, building social dialogue among stakeholders, and protecting worker rights [12]. Accordingly, decent work includes safe working conditions, fair pay, and access to healthcare [37]. Precarious work, in contrast, exposes workers to poor working conditions, poor salaries, restrictions on advocating workplace benefits [6], insecurity [38], and low control over working conditions [39]. Precarious work refers to any form of waged work identified with several precariousness dimensions [38, 40] and is, therefore, not restricted to nonstandard employment (such as temporary or contract work) or a specific employment type [41].

Precarious work has been defined as a construct comprising objective work characteristics, such as short-term contracts or low wages [6]. However, it has also been conceptualised as subjective experiences of work precarity (or perceived job precariousness) and refers to individual experiences of job insecurity, instability, and powerlessness related to one's work [42]. Separating objective and subjective work precariousness can be beneficial, as emergency nurses' subjective experiences of external conditions are potentially a better predictor of outcomes (such as capabilities or mental health) than objective ones [6].

Precarious work is essentially composed of the following work components: (a) nonstandard, atypical, alternative, nonregular, or contingent work; (b) low economic security due to insufficient remuneration; (c) limited power and control to workers; (d) poor workplace protection and rights; and (e) exposure to unsafe working conditions [6]. Consequently, individuals who experience precarious work are losing their jobs, fear losing their jobs, or lack alternative employment opportunities [5, 43]. Koranyi et al. [40] point out that precarious work entails accumulating unfavourable job quality dimensions. Therefore, precarious work is not necessarily the result of a single dimension; it is a multidimensional construct of employment insecurity, income inadequacy, and a lack of rights and protection [44]. Moreover, it extends beyond the confines of uncertainty about the continuity of one's work to the uncertainty of one's ability to cope with unexpected situations due to insufficient social power and resources [45].

Individuals evaluate their job quality according to their expectations and experiences, the standards of the community, and what they see from others' experiences [46]. Therefore, precarious work is characterised by a compilation of poor job quality dimensions such as temporary work, job insecurity, vulnerability, poor work control and employee rights, poor work scheduling, limited development opportunities, and a poor salary [34]. Moreover, precarious work is considered as nonstandard working arrangements that put workers and their families at risk, producing adverse health outcomes [47].

Creed et al. [42] argue that job precariousness consists of four factors: (a) perceived job conditions; (b) remuneration; (c) security; and (d) flexibility. A systematic review revealed that precariousness comprised poor salaries, poor working conditions, and job insecurity [44]. However, as precarious work is primarily the accumulation of poor job quality dimensions, emergency nurses' precarious work could focus on their experiences of job quality [41]. A high-quality job has a sufficient salary, benefits, good working conditions, and professional development opportunities [46]. Therefore, in this study, precarious work comprises four primary dimensions: (a) poor salary; (b) poor working conditions; (c) job insecurity; and (d) poor professional development.

Emergency nurses' sustainable employability depends on the opportunities available to them to pursue meaningful work while fostering good health and well-being [31] and having the freedom to choose a life they have reason to value [13]. Precarious work may have an impact on emergency nurses' capabilities and capability sets because it affects the enablement and achievement of valued aspects of their jobs [31]. Within the SE framework, constraints such as precarious work have the potential to have a negative impact on emergency nurses' capabilities [13]. Murangi et al. [20] found that precarious work negatively affected special school educators' capabilities, such as using and developing knowledge and skills, involvement in important decisions, building and maintaining meaningful relationships at work, setting their own goals, earning a good income, and contributing to something valuable.

### 2.3. Personal Resources: The Role of Mental Toughness

Mental toughness is a positive psychological resource that individuals apply to navigate several challenging and stressful situations. Mental toughness has been conceptualised as “a state-like psychological resource that is purposeful, flexible, and efficient in nature for the enactment and maintenance of goal-directed pursuits” ([26], p. 18.). It affects an individual's stress tolerance and ability to consistently perform at optimal capacity, regardless of the circumstances [48]. Therefore, it is a personal variable separate from process, outcome, task, relationship, or culture. As mental toughness is conceptualised as a resource, it forms part of a larger group of concepts. These concepts can either be centrally valued by the person (such as self-esteem or health) or represent a means to obtain valued ends (such as money or social support) [26].

Mental toughness draws from the three components of hardiness [49]: (a) challenge (the acceptance of stress and adversity as a part of life and a requirement for growth and development); (b) commitment (remaining in stressful situations that produce meaning); and (c) control (the capacity to remain true to influence outcomes). A meta-analysis on hardiness confirmed its ability to protect individuals from the adverse outcomes of stress regarding their performance and health [50]. Apart from the three concepts derived from hardiness, confidence is added to form mental toughness, distinguishing it from hardiness (see [26]). Mental toughness has been operationalised as a four-factor construct; however, it has also been found to be unidimensional [51].

A systematic review showed that mental toughness is associated with numerous positive psychological traits, improved coping mechanisms, and better mental health. Furthermore, it showed that mental toughness could be advantageous in various contexts (e.g., education, workplace, and military). Persons with high mental toughness are able to display confidence when faced with demanding situations, which contributes to psychological well-being. They are also more inclined to portray higher inhibitions to commit to their tasks and adopt problem-focused coping strategies for managing stress, such as motivational imagery and self-enhancing humour [52].

Emergency nurses work in a demanding environment where they are required to commit to tasks and be able to react to unique situations instantaneously. Therefore, their level of mental toughness can potentially serve as a personal resource, reducing the negative impact of precarious work on their capabilities and contributing to their mental health. Precarious work negatively affects the accumulation of work capabilities, which, in turn, influences mental health [20]. Mental toughness, as a resource, assists people in achieving goals, while navigating challenging and stressful situations. Therefore, within the sustainable employability framework, it serves as a personal resource, enabling emergency nurses to achieve valued work outcomes (i.e., work capabilities), despite precarious work [13]. For instance, precariousness regarding work context is expected to reduce the enablement and achievement of emergency nurses. Mental toughness can assist emergency nurses to persevere in achieving valued work outcomes when faced with adverse working conditions.

### 2.4. Mental Health as a Functioning

Within the sustainable employability framework, the capability set of emergency nurses affects their functionings such as mental health at work [13, 21]. Mental health has been conceptualised by Keyes [53] as a syndrome of symptoms comprising positive feelings and functionings in life. The mental health continuum [54, 55] suggests that although mental health and mental illness are related, they are distinct in that a lack of psychopathology does not solely produce positive mental health. A person presenting with positive mental health is described as flourishing, whereas a lack of mental health is viewed as languishing. Thus, mental health is expressed through a continuum between languishing and flourishing. Accordingly, poor mental health does not indicate the presence of mental ill-health (e.g., depression, posttraumatic stress disorder), although an extended state of languishing could potentially produce mental ill-health [56]. Also, flourishing (compared to languishing) individuals are more likely to recover from a mental illness [57]. Consequently, in this study, mental health is viewed as a continuum of positive mental health, varying from flourishing (i.e., a combination of feeling good about and functioning well in life) to languishing (i.e., not feeling good about and not functioning well in life) [58]. Negative affect, which indicates low mental health, is used as an additional indicator of (poor) mental health. Anger, sadness, anxiety, boredom, frustration, and guilt are common unpleasant emotions associated with adverse events and a lack of need for gratification [59].

Based on the conceptualisation of Keyes [53, 54, 58], Rautenbach and Rothmann [60] define mental health (varying from flourishing to languishing) at work as consisting of three dimensions: emotional, psychological, and social well-being. Emotional well-being refers to experiencing positive affect and job satisfaction. Emergency nurses with high emotional well-being generally exhibit positive emotions and are satisfied with their jobs. Psychological well-being entails autonomy, competence, relatedness, engagement, meaningful work (finding meaning in one's work), and learning. Finally, social well-being includes social acceptance, social actualisation, social coherence, social contribution, and social integration. Subsequently, emergency nurses with a high level of social well-being present with a high sense of being part of the hospital and contributing to its development and functioning, believing they are contributing towards something meaningful. Also, emergency nurses with a high sense of mental health at work will typically be able to positively assess their work and perform effectively with high productivity levels.

For emergency nurses to be sustainably employable, they need the freedoms and opportunities to enjoy personal, social, and environmental conditions, allowing them to make meaningful contributions to the world without putting their health and well-being at risk [31]. Therefore, they need to identify what they value and what matters at work to ultimately pursue a working life they have reason to value [13]. The first empirical study on work capabilities investigated three functionings of Dutch workers: (a) work-role functioning; (b) workability; and (c) work performance [31]. A study in Namibia showed that seven work capabilities had a positive impact on educators' emotional, psychological, and social well-being [20]. Similar results were found among South African secondary school teachers [61]. Thus, capabilities are vital in promoting mental health at work [20, 61]. Furthermore, within the sustainable employability framework, precarious work can negatively affect emergency nurses' mental health [35, 62]. Precariousness regarding work conditions and job insecurity were found to negatively influence emotional, psychological, and social well-being, while precarious professional development affected psychological and social well-being [20].

## 3. Current Study

Emergency nurses require capabilities to achieve valued work outcomes, affecting their mental health. However, external conditions such as precariousness can have an impact on these nurses' ability to realise capabilities (enablement and achievement of valued work aspects). The extent to which emergency nurses perceive their work as precarious (poor salary, poor working conditions, job insecurity, and poor professional development) can shape their capability set (accumulation of realised capabilities), affecting their mental health. Based on the sustainable employability model, personal factors can determine the conversion of available resources into capabilities. Consequently, emergency nurses' willingness and motivation to capitalise on their surroundings will have an impact on their capabilities. Therefore, the effects of precarious work on emergency nurses' capabilities will be lower for those with high (versus low) mental toughness levels.

Precarious work and mental toughness will affect emergency nurses' work capability set, influencing their mental health and, ultimately, contributing to the sustainability of their employment as emergency nurses. Therefore, the study aimed to investigate emergency nurses' precarious work and mental toughness within the South African context and the effect of these on their work capabilities and mental health at work.

**Figure 1:**
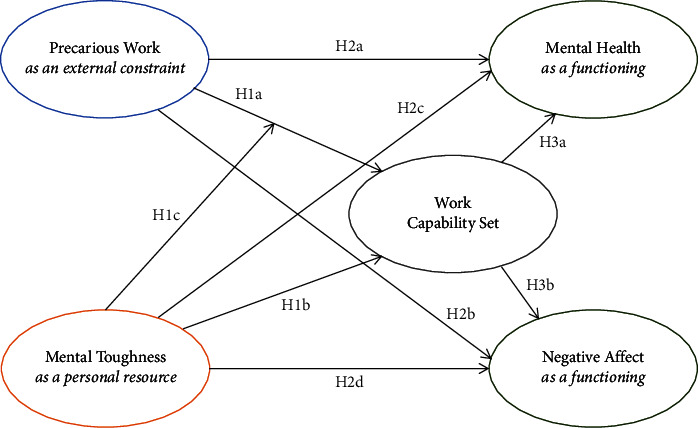
The effect of precarious work and mental toughness on capabilities, mental health, and negative affect. Note: H4 (not included in Figure 1) concerns the indirect effects of precarious work on mental health and negative affect via the work capability set.

The following hypotheses were set (see Figure 1):

  Hypothesis 1: Precarious work negatively affects emergency nurses' capability set (hypothesis 1a), while their mental toughness positively affects their capability set (hypothesis 1b). Also, emergency nurses' mental toughness interacts with their experiences of precarious work to affect their capabilities (hypothesis 1c).
  Hypothesis 2: Precarious work negatively affects emergency nurses' mental health at work (hypothesis 2a) and is associated with their negative affect (hypothesis 2b). Mental toughness is positively associated with mental health (hypothesis 2c) and negatively associated with negative affect (hypothesis 2d).
  Hypothesis 3: Emergency nurses' capability set is positively associated with their mental health (hypothesis 3a) and negatively associated with negative affect (hypothesis 3b).
  Hypothesis 4: Precarious work indirectly affects mental health (hypothesis 4a) and negative affect (hypothesis 4b) via the capability set of emergency nurses.

## 4. Methods

### 4.1. Participants

South Africa's healthcare system consists of public and private sectors that run parallel to each other. The government funds the public healthcare system, which serves close to 71% of the South African population. In comparison, the revenue of the private healthcare system is mostly from medical aid schemes and health insurance paid for by the individual [63]. The study surveyed persons working as nurses in the emergency department of hospitals providing Level 1 or 2 trauma care. A Level 1 trauma care hospital can provide leadership and total care for every aspect of injury (from prevention to rehabilitation) while providing 24-hour availability of all primary specialities and a trauma surgeon as director. A Level 2 trauma care hospital can provide 24-hour medical cover for initial definitive trauma care, regardless of injury severity (including the typical specialities) [64]. Permission was obtained from 13 private hospitals and one public hospital in the Gauteng province. The final sample consisted of 204 emergency nurses.

Table 1 shows that more females (71.57%) than males (26.47%) participated in the study. Most participants were aged between 30 and 39 years (28.43%), with a higher certificate (25.98%), a three-year diploma (24.02%), and a bachelor's degree (20.59%). Most nurses had between one and five years of emergency nursing experience (25.49%), followed by those in the category of six to 10 years (24.02%). A total of 75% of the participants were in permanent positions.

### 4.2. Measuring Instruments

Emergency nurses' perceptions of precariousness in their job were assessed using the *Precarity Position Profile* (PPP; [20]) questionnaire. The PPP consists of 16 items measuring four components: (a) salary (two items, e.g., “My current salary allows me to cover my basic needs.”); (b) work conditions (five items, e.g., “At work, I am treated in an unjust manner.”); (c) job insecurity (six items, e.g., “I feel insecure about the future of my job.”); and (d) professional development (three items, e.g., “I am able to advance my knowledge and skills at work.”). The items were rated on a Likert-type scale, ranging from 1 (*never*) to 5 (*always*). In a study among Namibian special education teachers, Murangi et al. [20] found the PPP to be reliable (*ω* = 0.61 to 0.79) and valid.

Emergency nurses' mental toughness was investigated via the *Mental Toughness Questionnaire*-*Short Form* (MTQ-10; [65]. The MTQ-10 consists of 10 items rated on a five-point Likert-type scale (1 = *strongly disagree* to 5 = *strongly agree*). An example of an item is “Even when under considerable pressure, I usually remain calm.” Papageorgiou et al. [65] reported test-retest reliability (*α* = 0.74 and *α* = 0.75), and Dagnall et al. [51] also reported satisfactory reliability in their study (*α* = 0.77).

The *Capability Set for Work Questionnaire* (CSWQ; [31] was used to measure emergency nurses' work capabilities. The CSWQ measures seven predetermined work values: (a) using knowledge and skills; (b) developing knowledge and skills; (c) involvement in important decisions; (d) building and maintaining meaningful relationships at work; (e) setting own goals; (f) earning a good income; and (g) contributing to something valuable. For each of these seven values, the emergency nurses were requested to indicate whether (a) they considered the work value important (importance: e.g., “How important is it to you to be able to use your knowledge and skills at work?”), (b) their work was offering them sufficient opportunities to achieve it (enablement: e.g., “Does your current work offer you enough opportunities to do that?”), and (c) they succeeded in achieving it (achievement: e.g., “To what extent do you succeed in doing so?”). The items were rated on a Likert scale, ranging from 1 (*totally not*) to 5 (*to a very great extent*). The CSWQ has convergent, predictive, and incremental validity [66] and is reliable (*ω* = 0.77) [20, 67].

The *Flourishing-at-Work Scale*-*Short Form* (FAWS-SF; [60] was administered to measure emergency nurses' workplace mental health. The FAWS-SF consists of 17 items that measure an employee's well-being on a continuum that ranges from languishing to flourishing. The scale items are scored on a six-point Likert-type scale, ranging from 1 (*never*) to 6 (*every day*), indicating the frequency with which respondents experience each identified symptom of well-being. The FAWS-SF consists of three scales, namely, (a) emotional well-being (three items, e.g., “During the past month at work, how often did you experience satisfaction with your work?”), (b) psychological well-being (nine items, e.g., “During the past month at work, how often did you feel that the work you do serves a greater purpose?), and (c) social well-being (five items, e.g., “During the past month at work, how often did you feel this organisation is becoming a better place for people like you?”). In a study by Rautenbach and Rothmann [60]; the reliability coefficients of all the scales were acceptable (*ρ* ≥ 0.70). Scale reliabilities ranged between 0.75 and 0.95, indicating acceptable internal consistency [68]. The findings of their validation study support the construct validity and internal consistency of the FAWS-SF. Murangi et al. [20] found acceptable McDonald's omega coefficients for the three dimensions among Namibian special education teachers (*ω* = 0.80 to 0.88).

### 4.3. Research Procedure

The North-West University Health Research Ethics Committee (NWU-HREC) provided ethics clearance for the study (NWU-00273-21-A1). Following the granting of ethics clearance for the study, the researcher obtained permission from four private hospital groups, the corresponding hospitals, and emergency department management. Additionally, the Provincial Department of Health and the participating public hospital gave permission for the study. Data was collected via an online platform (i.e., QuestionPro) and hard-copy booklets. The researcher explained the research purpose, that participation in the study was voluntary, and that all information and responses would be kept confidential and anonymous. Participation was predominately through hard-copy booklets (91.18%).

### 4.4. Data Analysis

Data analyses were performed using SPSS Version 27 [69] and Mplus Version 8.8 [70]. Structural equation modelling (SEM) was performed to test measurement and structural models. The robust weighted least squares (WLSMV) estimator was used to perform confirmatory factor analysis (CFA). Model fit was assessed through multiple goodness-of-fit indices and information criteria to select the best model fit for the data [71]: the chi-square statistic (the test of absolute fit of the model), standardised root mean residual (SRMR), root mean square error of approximation (RMSEA), Tucker–Lewis index (TLI), and comparative fit index (CFI). A TLI and CFI score higher than 0.90 indicates an acceptable value, with a score higher than 0.95 indicating an excellent fit. For SRMR and RMSEA values to be acceptable, a score below 0.08 is required with a 90% confidence interval, not including zero [72]. Scale reliability was investigated using the McDonald's omega coefficient (*ω*).

Pearson correlations were used to investigate the relationships between precarious work, mental toughness, and mental health [73]. Point biserial correlations were used in determining the associations of work capabilities with precarious work, mental toughness, and mental health at work. An analysis of the effect of work precariousness on the capabilities of emergency nurses moderated by mental toughness was conducted using PROCESS Version 4.0 (model number 1) [74] in SPSS Version 27 [69]. The focal predictor's conditional effect at the moderator's values was investigated using the Johnson-Neyman (JN; [75]) technique. When a moderator (i.e., mental toughness) has a significant effect on a dependent variable (the capability set), the JN method is used to determine the values for which the moderator is significant [76].

Multivariate analysis of variance (MANOVA) was used to investigate the difference in precarious work among emergency nurses based on biographical information. Jamovi Version 2.3 [77] was used to conduct the analysis.

## 5. Results

### 5.1. Testing the Measurement Model

Confirmatory factor analysis (CFA) was used to test one- and four-factor measurement models of precarious work and one-, two-, and three-factor measurement models of mental health (i.e., flourishing at work). The respective survey items were the latent variable indicators of the model, which were as follows: (a) precarious work: salary (two items), work conditions (five items), job insecurity (six items), and professional development (three items); (b) mental toughness (six items); (c) the work capability set consisting of using knowledge and skills, developing knowledge and skills, involvement in important decisions, meaningful relationships at work, earning a good income, setting own goals, and contributing to something valuable (three items each); (d) mental health, consisting of emotional well-being (four items), psychological well-being (nine items), and social well-being (five items); and (e) negative affect (three items). The fit statistics of the measurement models are reported in Table 2.

Three precarious work measurement models were tested, namely; (a) a four-factor model; (b) a one-factor model; and (c) a four-factor model with work precariousness as a second-order factor. From Table 2, it is evident that a model consisting of four correlated factors fitted the data best. Allowing for a second-order factor (work precariousness) did not significantly improve the model.

The tests of the competing measurement models were performed based on precarious work (four first-order factors: salary, work conditions, job insecurity, and professional development), mental toughness (one first-order factor), capability set (one first-order factor), and mental health (three second-order factors: emotional well-being, psychological well-being, and social well-being). Confirmatory factor analysis showed that the four negatively phrased mental toughness items did not load sufficiently on mental toughness. Consequently, it was decided to remove the following four negatively phrased items from the model: item 2 (“I tend to worry about things well before they actually happen.”), item 3 (“I usually find it hard to summon enthusiasm for the tasks I have to do.”), item 6 (“I just don't know where to begin is a feeling I usually have when presented with several things to do at once.”), and item 7 (“When I make mistakes, I usually let it worry me for days after.”). The fit statistics of the measurement model that fitted the data best were as follows: *χ*^2^ = 1697.53 (d*f* = 1144, *p* < 0.001); RMSEA = 0.05 [0.04, 0.05], *p*=0.666; CFI = 0.95; TLI = 0.94; SRMR = 0.07. All the fit indices showed acceptable fit compared to the cut-off values. The sizes of the factor loadings of the items on their target factors were acceptable: salary: *λ* = 0.78 to 0.97 (mean = 0.88); work conditions: *λ* = 0.60 to 0.86 (mean = 0.79); job insecurity: *λ* = 0.66 to 0.84 (mean = 0.74); professional development: *λ* = 0.70 to 0.95 (mean = 0.81); capability set: *λ* = 0.67 to 0.87 (mean = 0.79); mental toughness: *λ* = 0.56 to 0.83 (mean = 0.68); emotional well-being: *λ* = 0.75 to 0.83 (mean = 0.78); psychological well-being: *λ* = 0.69 to 0.86 (mean = 0.76); social well-being: *λ* = 0.81 to 0.92 (mean = 0.86); and negative affect: *λ* = 0.75 to 0.98 (mean = 0.83). Therefore, the factors were well-defined and aligned with theoretical expectations.

### 5.2. Construct Validity of the Precarity Position Profile

The construct validity of the Precarity Position Profile (PPP) instrument was investigated using confirmatory factor analysis. The result of the analysis is reported in Table 3.

As shown in Table 3, all factor loadings were significant (*p* < 0.001 for all items). The standardised factor loadings for all the items were above the suggested value of 0.50 [80], with most above 0.70. The omega reliability of the four factors of precarious work were as follows: 0.81 (salary), 0.86 (work conditions), 0.81 (job insecurity), and 0.81 (poor professional development).

### 5.3. Descriptive Statistics, Reliabilities, and Correlations

The McDonald's omega reliabilities, means, standard deviations, and Pearson correlations of the variables used in the study are reported in Table 4. McDonald's omega coefficients above 0.70 were obtained for all the scales, indicating acceptable reliability [68].

Table 4 shows that all four precarious work components had a significant (*p* < 0.01) association with emergency nurses' capability set (medium effects), of which professional development was the strongest (*r* = −0.49). Emergency nurses' mental toughness and capabilities were significant and positively related (medium effect). Regarding their functioning, emergency nurses' capability set had a significant relationship with their mental health constituents. Capabilities were positively associated with emotional, psychological, and social well-being (large effects) and negatively with negative affect (medium effect).

Not shown in Table 4 are the associations between precarious work and capabilities. Except for precariousness about salary with using and developing knowledge and setting own goals and precariousness about work conditions with using knowledge and skills and setting own goals, all the correlations were statistically significant (*p* < 0.01).

The difference in emergency nurses' precarious work based on their gender and level of education was investigated through a multivariate analysis of variance (MANOVA). No statistically significant differences were found between precarious work experiences (i.e., salary, work conditions, job insecurity, and professional development) of different genders and education groups.

Regarding emergency nurses' functioning, all the capabilities had significant (*p* < 0.01) relationships (medium effect) with their emotional, psychological, and social well-being, apart from setting own goals (small effect). Emotional well-being was associated with using (*r* = 0.35) and developing (*r* = 0.34) knowledge and skills, involvement in important decisions (*r* = 0.37), meaningful relationships at work (*r* = 0.32), earning a good income (*r* = 0.40), and contributing to something valuable (*r* = 0.38). Psychological well-being was related to using (*r* = 0.34) and developing (*r* = 0.35) knowledge and skills, being involved in important decisions (*r* = 0.30), meaningful relationships at work (*r* = 0.35), earning a good income (*r* = 0.37), and contributing to something valuable (*r* = 0.37). Social well-being was associated with using (*r* = 0.34) and developing (*r* = 0.36) knowledge and skills, being involved in important decisions (*r* = 0.35), building and maintaining meaningful relationships at work (*r* = 0.33), earning a good income (*r* = 0.38), and contributing to something valuable (*r* = 0.42).

The three highest significant (*p* < 0.01) relationships between negative affect and work capabilities were earning a good income (*r* = −0.29), being involved in important decisions (*r* = −0.23), and using knowledge and skills (*r* = −0.21).

### 5.4. Testing the Structural Model

Confirmatory factor analysis was used to test the structural model of precarious work, mental toughness, capabilities, and mental health (see Table 5).

From Table 5, it is evident that emergency nurses' precariousness regarding their salary (*β* = −0.31, *p* < 0.001), work conditions (*β* = −0.24, *p* < 0.014), and professional development (*β* = −0.26, *p* < 0.001) had negative effects on their capability set, while mental toughness had a positive effect (*β* = 0.26, *p* < 0.003). One reason for the insignificant relationship between job insecurity (as a component of precarious work) and the capability set in the structural model, was the strong correlation between precariousness about work conditions and job insecurity (*r* = 0.59, *p* ≤ 0.001). Therefore, hypotheses 1a, 1b, and 1c were accepted. Furthermore, emergency nurses' precariousness regarding their work conditions (*β* = −0.35, *p* < 0.001) and professional development (*β* = −0.28, *p* < 0.001) had a negative impact on their mental health, while mental toughness (*β* = 0.33, *p* < 0.001) and the capability set (*β* = 0.28, *p* < 0.001) positively influenced their mental health. Finally, the precarious work conditions (*β* = 0.35, *p* < 0.001) affected emergency nurses' negative affect. Hypothesis 2a was accepted for three dimensions of the impact of precarious work on mental health: (a) salary, (b) work conditions, and (c) professional development. Hypothesis 2b was only accepted for the effect of precarious work conditions on negative affect. Hypothesis 2c was accepted, while hypothesis 2d was rejected. Hypothesis 3a was accepted, while hypothesis 3b was rejected.

**Figure 2:**
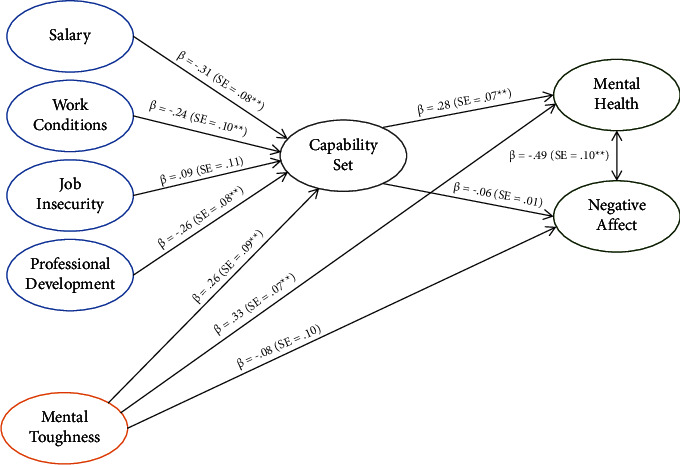
Structural model of precarious work, mental toughness, capabilities, mental health, and negative affect. Notes: *β* = regression coefficient; SE = standard error; ^*∗∗*^*p* < 0.01.

Figure 2 shows the structural model of emergency nurses' precarious work, mental toughness, capabilities, mental health, and negative affect.

As depicted in Figure 2, low precariousness (regarding salary, work conditions, and professional development) and mental toughness positively affected emergency nurses' capability set (*R*^2^ = 0.58, large effect). Furthermore, low precariousness about work conditions and professional development, mental toughness, and a strong capability set were the best predictors of mental health (*R*^2^ = 0.38, large effect).

### 5.5. Moderating Effects

With the capability set as the dependent variable, the four factors of precarious work (predictors) were entered in the first step, followed by mental toughness (moderator) in the second step. Interaction scores between precarious work factors and mental toughness were entered in the third and final step to examine the possibility of a moderating effect. A significant interaction term between a predictor and a moderator indicates a moderating effect [76].

**Figure 3:**
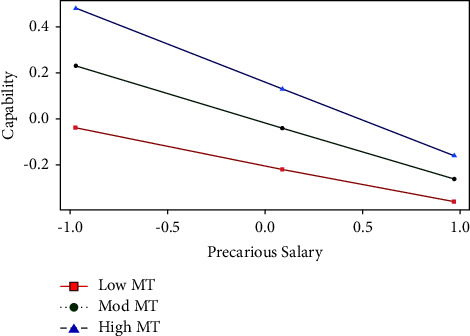
Interaction: precariousness about salary and mental toughness (MT).

The interaction between salary (as a factor of work precariousness) and mental toughness (beta = −0.17, *p*=0.049 [−0.34, 0.00]) accounted for a significant addition of 1% in the variance of the capability set (*F* (6, 197) = 29.59, *p* < 0.001). Precariousness about salary interacted with mental toughness to affect the capability set. To examine the interaction effects that emerged, the simple slopes of the precariousness about salary–mental toughness at the 16th, 50th, and 84th percentiles [76] were inspected. We also tested whether each slope was statistically significant for the moderating effect (see Figure 3).

Figure 3 shows that the three lines in the figure have different slopes. Each of these lines reflects the conditional effects of mental toughness on the capability set. A simple slope analysis showed that precariousness about salary had a statistically significant effect on the capability set among those with low (value = 0.52, effect = −0.17, *t* = −2.49, *p*=0.014 [−0.30, −0.03]), moderate (value = −0.02, effect = −0.25, *t* = −5.54, *p* < 0.001 [−0.34, −0.16]), and high (value = 0.45, effect = −0.33, *t* = 0.06, *p* < 0.001 [−0.44, −0.22]) scores on mental toughness. The steepest slope (the strongest positive association between low precariousness about salary and the capability set) occurs for individuals who reported high mental toughness. In contrast, the flattest slope (the weakest association) occurs for individuals who reported low mental toughness. The weakest association between low precariousness about salary and the capability set occurred for emergency nurses who reported low mental toughness. Therefore, mental toughness strengthens the association between low precariousness about salary and the capability set.

**Figure 4:**
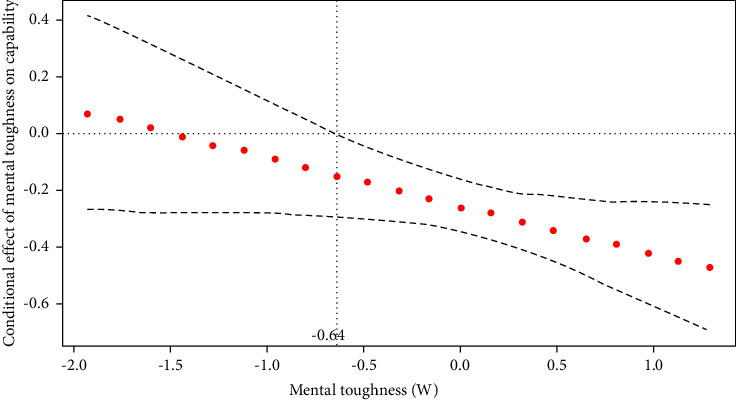
Conditional effect of mental toughness on salary precariousness on the capability set.

Probing the interaction using the JN technique (see Figure 4) shows that the conditional effect of precariousness about salary on the capability set was statistically significant across 88.73% of the scores on the moderator (i.e., mental toughness). The analysis identified a mental toughness score of −0.64 as the transition point. The predicted value of the capability set was statistically significant between low and high precariousness about salary at mental toughness scores above −0.64. Therefore, hypothesis 1c was accepted for precariousness about salary.

### 5.6. Mediating Effects

Next, the indirect effects of precarious work and mental toughness on mental health and negative affect (via the capability set) were computed using the procedure suggested by Hayes [76].

The significant indirect negative effects of three precarious work dimensions on mental health via a poor capability set included: (a) salary (*β* = −0.09 [−0.21, −0.04]), (b) work conditions (*β* = −0.07 [0.17, −0.02]), and (c) professional development (*β* = −0.08 [−0.17, −0.03]). Also, mental toughness had a significant positive indirect effect on mental health via a strong capability set (*β* = 0.07 [0.02, 0.16]). None of the indirect effects of precarious work and mental toughness on mental health and negative affect were significant. Hypothesis 4 was accepted only for the indirect effects of three precariousness dimensions (salary, work conditions, and professional development) on mental health via the capability set of emergency nurses.

In conclusion, it is evident that emergency nurses' work capabilities are negatively associated with precarious work. Hypothesis 1a is therefore accepted in that an increase in emergency nurses' perceptions of their work being precarious will decrease the realisation of their work capabilities (achievement and enablement of work values). There was a positive relationship between emergency nurses' mental toughness and their work capabilities. Hypothesis 1b is thus accepted in that an increase in their mental toughness is associated with increased work capabilities. Mental toughness moderated the relationship between emergency nurses' precariousness about salary and their work capability set. The hypothesis (H1c) that emergency nurses' mental toughness interacts with their experiences of precarious work to affect their capabilities is therefore accepted for precarious salary.

Three precarious work components (i.e., salary, work conditions, and professional development) had a negative association with emergency nurses' mental health at work. In comparison, only precariousness about work conditions had a negative relationship with their negative affect. Furthermore, mental toughness had a positive relationship with mental health. Consequently, the hypothesis that an increase in precarious work will decrease emergency nurses' mental health is accepted for salary, work conditions, and professional development (H2a), and work conditions for negative affect (H2b). The hypothesis that an increase in emergency nurses' mental toughness will increase their mental health is accepted (H2c). The hypothesis (H2d) that increased mental toughness will decrease emergency nurses' negative affect is rejected as the relationship between the two variables was insignificant.

Emergency nurses' capability set was positively associated with their mental health, and the relationship between their capability set and negative affect was insignificant. Therefore, hypothesis 3a, that an increase in emergency nurses' capability set will increase their mental health, is accepted. However, the hypothesis (H3b) that it will decrease their negative affect is rejected.

Only precariousness about salary, work conditions, and professional development indirectly affected emergency nurses' mental health through their work capability set. However, the indirect effect of precarious work on their negative affect via the work capability set was insignificant. Thus, the hypothesis that precarious work indirectly affects mental health via their work capability set (H4a) is accepted, while the hypothesis that it indirectly affects their negative affect (H4b) is rejected.

## 6. Discussion

This study investigated emergency nurses' sustainable employability in South Africa from the perspective of precarious work, mental toughness, work capabilities, and mental health. The results confirmed that the work precariousness of emergency nurses was indeed negatively associated with their work capabilities. Emergency nurses' capability set positively affected their mental health, with mental toughness moderating the effect of poor salary (a component of precarious work) on capabilities. Precariousness regarding salary, work conditions, and professional development affected emergency nurses' mental health indirectly and negatively through a poor capability set, while mental toughness indirectly and positively affected their mental health through a strong capability set.

Three precarious work dimensions (i.e., salary, work conditions, and professional development) were negatively associated with emergency nurses' capabilities and mental health. In line with the findings of previous studies (e.g., [20, 31]) and the conceptualisation of SE [13], emergency nurses' capability set and mental toughness affected their mental health at work. Mental toughness moderated the negative impact of precariousness caused by a poor salary on their capabilities.

Conditions of injustice (arising from precarious work) can restrict and damage the capability development of individuals [19], negatively affecting their mental health. Sen [23, 25] articulates the connection between social justice and well-being in the CA. Social justice constitutes individuals' opportunities to achieve valued beings and doings, e.g., work capabilities [27]. Although inner resources (e.g., mental toughness) may impact individuals' capabilities, external conditions of justice play a vital role in capability development. Therefore, individuals must feel secure and free to develop their capabilities [23, 24].

The structural model of mental health showed that precariousness regarding salary, work conditions, and professional development was significantly and negatively associated with emergency nurses' work capability set, which confirms the findings of Allan et al. [6], Morphet et al. [7], and Murangi et al. [20]. Emergency nurses who experience their work as precarious may not have the opportunity to develop their capabilities because they have a short time perspective (and, therefore, limited time and space to consider their future) and little control over their life choices [24].

Three precarious work dimensions and the emergency nurses' capability set impacted their mental health. Regarding emergency nurses' functioning at work, all capabilities were positively related to their emotional, psychological, and social well-being. These results confirm the findings of De Wet and Rothmann [61] among secondary school educators in South Africa. Furthermore, three work capabilities, namely earning a good income, involvement in important decisions, and using knowledge and skills, were associated with the reduced negative effect of emergency nurses. Negative affect indicates low mental health [58] and is associated with a lack of need fulfilment [59].

As a personal resource, emergency nurses' mental toughness directly shaped their capabilities and mental health (specifically emotional, psychological, and social well-being). An additional 1% of the variance in emergency nurses' capability sets was explained through the interaction between their precariousness about salary and mental toughness. Low precariousness about salary mattered most for the capability set when mental toughness was moderate to high. Emergency nurses' mental toughness strengthened the effect of low salary precariousness on their capability set. Therefore, it was evident that moderate to high scores on mental toughness (as a personal resource) moderated the effect of emergency nurses' precariousness about salary on their work capabilities [52]. Their mental toughness (as a personal resource) assisted them in achieving work capabilities when precariousness about salary was low. Negative affect was not significantly associated with mental toughness and the capability set.

Precariousness regarding salary, work conditions, and professional development affected emergency nurses' mental health indirectly and negatively through a poor capability set, while mental toughness indirectly and positively affected their mental health through a strong capability set. Consequently, emergency nurses with mental toughness and a comprehensive capability set are expected to present with higher mental health at work, lessening the impact of precarious work on it, especially when perceiving their salary, work conditions, and professional development as poor [13, 52].

The results of this study support the value of the CA [23, 25, 28] and, specifically, the sustainable employability model [13, 27, 31] for understanding the effects of precarious work and mental toughness on emergency nurses' capabilities and mental health (emotional well-being, psychological well-being, social well-being, and negative affect). Indeed, work can be better conceptualised as a means of flourishing when emergency nurses' values are incorporated into evaluations of their employment [30].

The findings of this study contribute to the literature in the following ways. First, it showed that precariousness regarding salary, work conditions, and professional development impacted emergency nurses' capability set, which, in turn, affected their mental health at work. Emergency nurses' mental toughness (as a personal resource) assisted them in achieving valued work outcomes and contributed to their mental health. Second, the study produced new information on the associations among precarious work components, mental toughness, capabilities, and mental health of emergency nurses. Third, the study showed that high mental toughness moderated the negative effects of precariousness regarding salary on emergency nurses' capabilities. Fourth, the results of this study confirmed that precariousness regarding salary, work conditions, and professional development affected emergency nurses' mental health indirectly and negatively through a poor work capability set.

### 6.1. Practical Implications

Policymakers should address employees' precariousness at work and assist them in developing work capabilities to improve their mental health, contributing to their sustainable employability [13]. Interventions directed at emergency nurses' precariousness regarding their salaries, work conditions, and professional development should be implemented. The CA offers a useful framework to address precariousness at work [4]. Therefore, interventions need to focus on improving emergency nurses' support and resources to reduce their precariousness at work. For example, emergency nurses' precariousness regarding their salary could be improved by re-evaluating current financial and nonfinancial reward policies to determine the best possible reward models for these professionals. Given that emergency nurses' mental toughness is related to their capabilities and mental health, research and interventions focusing on the personal resources of nurses should be conducted [51]. Mental toughness can be improved through individual interventions (such as visualisations and affirmations) or, more complexly, mentor-mentee interventions (such as reality training and stress acclimatisation) (see [79]). Interventions should be implemented to decrease precarious work, assist them in achieving work capabilities, and improve their mental health [13, 27, 31].

### 6.2. Limitations and Recommendations for Future Research

This study had limitations that need to be acknowledged. First, the sample was drawn from a single province (i.e., Gauteng–the largest economic hub), mainly in the private sector. Future research, including different samples (e.g., other provinces and the public sector), could strengthen the generalisability of the results. Second, while the work values included in this study were considered sufficient [20, 31, 67], unique work values might exist in a South African context. Therefore, qualitative studies of the work values of emergency nurses are essential. Third, although the results supported the PPP as acceptable in measuring emergency nurses' precariousness at work, a larger, more diverse group across different demographics and occupations over time could add further evidence regarding the reliability and validity of the instrument.

## 7. Conclusion

Precarious work negatively affected emergency nurses' capabilities, negative affect, and emotional, psychological, and social well-being (as dimensions of positive mental health). Emergency nurses' mental toughness affected their capabilities and mental health positively. Specific dimensions of precarious work that impacted individuals' capabilities and positive mental health were salary, work conditions, and professional development, while only work conditions influenced negative affect. Furthermore, mental toughness and capabilities, such as using knowledge and skills, developing new knowledge and skills, being involved in decision-making, developing and maintaining meaningful work relations, setting own goals, earning a good income, and contributing something valuable, mediated the relation between work precariousness and emergency nurses' positive mental health. A lack of capabilities, such as earning a good income, being involved in important decisions, and using knowledge and skills, was associated with negative affect.

## Figures and Tables

**Table 1:** Characteristics of participants (*N* = 204).

Demographic variable	Grouping	*n*	%
Gender	Male	54	26.47
	Female	146	71.57
	Missing values	4	1.96

Age	18–29 years of age	27	13.24
	30–39 years of age	58	28.43
	40–49 years of age	30	14.71
	50–59 years of age	18	8.82
	60+ years of age	2	0.98
	Missing values	69	33.82

Highest qualification	Grade 12 (NQF 4)	19	9.31
	Higher certificate (NQF 5)	53	25.98
	Three-year diploma (NQF 6)	49	24.02
	Bachelor's degree (NQF 7)	42	20.59
	Honours degree (NQF 8)	28	13.73
	Master's degree (NQF 9)	1	0.49
	Other	5	2.45
	Missing values	7	3.43

Emergency nursing experience	Less than 1 year	8	3.92
	1–5 years	52	25.49
	6–10 years	49	24.02
	11–20 years	24	11.76
	21–30 years	8	3.92
	31+ years	1	0.49
	Missing values	62	30.39

Contract type	Permanent contract	153	75.00
	Fixed-term contract	17	8.33
	Agency placement	30	14.71
	Missing values	4	1.96

**Table 2:** Measurement models of precarious work, mental toughness, capabilities, mental health, and negative affect.

Model	*χ* ^2^	d*f*	CFI	TLI	RMSEA	95% CI	SRMR
One-factor	3777.26^*∗∗*^	1175	0.74	0.73	0.10	[0.10, 0.11]	0.125
Precarious work: 1-Factor	2226.25^*∗∗*^	1162	0.90	0.89	0.07	[0.06, 0.07]	0.09
Precarious work: 4-Factor (a)	**1697.53** ^*∗∗*^	**1144**	**0.95**	**0.94**	**0.05**	**[0.04**, **0.05]**	**0.07**
Precarious work: 4-Factor (b)	1769.32^*∗∗*^	1158	0.94	0.94	0.05	[0.05, 0.06]	0.08
Mental health: 1-Factor	1824.48^*∗∗*^	1147	0.93	0.93	0.05	[0.05, 0.06]	0.08
Mental health: 2-Factor	1770.20^*∗∗*^	1139	0.94	0.93	0.05	[0.05, 0.06]	0.07
Mental health: 3-Factor (a)	1645.16^*∗∗*^	1130	0.95	0.94	0.05	[0.04, 0.05]	0.07
Mental health: 3-Factor (b)	**1697.53** ^*∗∗*^	**1144**	**0.95**	**0.94**	**0.05**	**[0.04**, **0.05]**	**0.07**

*Notes*. *χ*^2^ = chi-square; d*f* = degrees of freedom; CFI = comparative fit index; TLI = Tucker–Lewis index; RMSEA = root mean square error of approximation; CI = confidence interval; SRMR = standardised root mean square residual; ^*∗∗*^*p* < 0.01; precarious work: 4-factor (a) = four first-order factors, (b) = four second-order factors; mental health: 3-factor (a) = three first-order factors, (b) = three second-order factors. The bold values highlight the precarious work and mental health measurement models that were selected for the purposes of the study (i.e., precarious work = four first-order factor structure and mental health = three second-order factor structure).

**Table 3:** Precarity position profile factor loadings.

Factor	Item text	*λ*	SE
Salary	My current salary allows me to cover my basic needs	0.97	0.10
	My current salary allows me to cover unexpected expenses	0.78	0.09

Work conditions	At work, I feel afraid to demand better working conditions	0.60	0.06
	I feel defenceless towards unfair treatment by my superiors at work	0.84	0.04
	At work, I am treated in an unjust manner	0.79	0.05
	At work, my superior makes me feel that I can be easily replaced	0.85	0.03
	I cannot freely express my views at work	0.86	0.03

Job insecurity	Chances are, I will soon lose my job	0.84	0.04
	I am sure I can keep my job	0.75	0.05
	I feel insecure about the future of my job	0.69	0.05
	I think I might lose my job in the near future	0.78	0.04
	I think I will not be relevant to my work in the near future	0.72	0.05
	I think I will not be able to find another job in the near future	0.66	0.07

Professional development	I am able to advance my knowledge and skills at work	0.70	0.06
	I am afforded the time and resources to further my knowledge and skills at work	0.78	0.05
	The job gives me a chance to use my personal initiative or judgement in engaging in developmental activities of my choice	0.95	0.05

*Notes*. *λ* = standardised factor loadings; SE = standard error; all *p* < 0.001.

**Table 4:** Descriptive statistics, reliabilities, and correlations of the scales.

Variable	*ω*	Mean	SD	1	2	3	4	5	6	7	8	9
(1) Salary	0.81	3.79	1.04	—	—	—	—	—	—	—	—	—
(2) Work conditions	0.86	2.65	1.04	0.31^*∗∗*^	—	—	—	—	—	—	—	—
(3) Job insecurity	0.81	2.11	0.86	0.23^*∗∗*^	0.68^*∗∗*^	—	—	—	—	—	—	—
(4) Professional development	0.81	2.65	1.02	0.22^*∗∗*^	0.37^*∗∗*^	0.52^*∗∗*^	—	—	—	—	—	—
(5) Capability set	0.82	0.50	0.33	−0.44^*∗∗*^	−0.41^*∗∗*^	−0.41^*∗∗*^	−0.49^*∗∗*^	—	—	—	—	—
(6) Mental toughness	0.79	4.00	0.64	−0.02	−0.12	−0.43^*∗∗*^	−0.32^*∗∗*^	0.39^*∗∗*^	—	—	—	—
(7) Emotional well-being	0.83	4.23	1.14	−0.30^*∗∗*^	−0.48^*∗∗*^	−0.45^*∗∗*^	−0.54^*∗∗*^	0.61^*∗∗*^	0.48^*∗∗*^	—	—	—
(8) Psychological well-being	0.89	4.66	0.93	−0.21^*∗∗*^	−0.46^*∗∗*^	−0.44^*∗∗*^	−0.52^*∗∗*^	0.59^*∗∗*^	0.55^*∗∗*^	0.92^*∗∗*^	—	—
(9) Social well-being	0.92	3.91	1.40	−0.26^*∗∗*^	−0.52^*∗∗*^	−0.42^*∗∗*^	−0.56^*∗∗*^	0.60^*∗∗*^	0.44^*∗∗*^	0.87^*∗∗*^	0.84^*∗∗*^	—
(10) Negative affect	0.84	2.82	1.38	0.26^*∗∗*^	0.48^*∗∗*^	0.38^*∗∗*^	0.34^*∗∗*^	−0.33^*∗∗*^	−0.18^*∗∗*^	−0.64^*∗∗*^	−0.55^*∗∗*^	−0.58^*∗∗*^

*Notes*. Positively worded items on salary and job insecurity were reverse-scored when dimensions were created; *ω* = McDonald's omega coefficient; SD = standard deviation; ^*∗∗*^*p* < 0.01; *r* < 0.30 = small effect; 0.30 < *r* < 0.50 = medium effect; *r* > 0.50 = large effect.

**Table 5:** Structural model of precarious work, mental toughness, capabilities, mental health, and negative affect.

Dependent variable	Independent variable	*β*	SE	EST	*p* values
Capability set	Salary	−0.31	0.08	−3.86	<0.001^*∗∗*^
	Work conditions	−0.24	0.10	−2.43	0.014^*∗∗*^
	Job insecurity	0.09	0.11	0.81	0.416
	Professional development	−0.26	0.08	−3.19	0.001^*∗∗*^
	Mental toughness	0.26	0.09	2.97	0.003^*∗∗*^

Mental health	Salary	0.01	0.06	−0.14	0.888
	Work conditions	−0.35	0.08	−4.41	<0.001^*∗∗*^
	Job insecurity	0.14	0.10	1.34	0.181
	Professional development	−0.28	0.08	−3.80	<0.001^*∗∗*^
	Mental toughness	0.33	0.07	4.59	<0.001^*∗∗*^
	Capability set	0.28	0.07	4.09	<0.001^*∗∗*^

Negative affect	Salary	0.08	0.08	1.01	0.313
	Work conditions	0.35	0.09	3.70	<0.001^*∗∗*^
	Job insecurity	0.00	0.10	0.01	0.992
	Professional development	0.12	0.10	1.25	0.213
	Mental toughness	−0.08	0.10	−0.91	0.363
	Capability set	−0.06	0.01	−0.52	0.602

*Notes*. *β* = standardised regression coefficient; SE = standard error; EST = estimate; ^*∗∗*^*p* < 0.01.

## Data Availability

The datasets generated for this study can be found in Rothmann, Sebastiaan; Barnard, Neil [80]; “Precarious Work, Mental Toughness, Capabilities, and Mental Health,” Mendeley Data, V1, doi: 10.17632/7cb8t29stj.1

## Additional Points

The article forms part of a research focus under Optentia Research Unit's Human Flourishing in Institutions Research Programme. The research focus is on work capabilities (enabled and achieved work values) and functionings at work (such as burnout, mental health, work engagement, and intention to leave), as well as precariousness. To ensure collaboration, comparison, transferability, consistency, and reusability, the measurement instruments, conceptualisations, and wording of the constructs/phenomena within this research focus have been standardised. Therefore, while the constructs and research approach of this article might share similarities with other research articles within this focus area (such as [20, 67, 80, 81]), the aim, population, and results are different and make separate, unique contributions.
